# The Association between DRD3 Ser9Gly Polymorphism and Depression Severity in Parkinson's Disease

**DOI:** 10.1155/2019/1642087

**Published:** 2019-04-15

**Authors:** Yan Zhi, Yongsheng Yuan, Qianqian Si, Min Wang, Yuting Shen, Lina Wang, Hui Zhang, Kezhong Zhang

**Affiliations:** ^1^Department of Neurology, The First Affiliated Hospital of Nanjing Medical University, No. 300 Guangzhou Road, Nanjing 210029, China; ^2^Department of Radiology, The First Affiliated Hospital of Nanjing Medical University, No. 300 Guangzhou Road, Nanjing 210029, China

## Abstract

More and more evidence suggests that dopamine receptor D3 gene (DRD3) plays an important role in the clinical manifestations and the treatment of Parkinson's disease (PD). DRD3 Ser9Gly polymorphism is the most frequently studied variant point. Our aim was to investigate the potential effect of DRD3 Ser9Gly polymorphism on modulating resting-state brain function and associative clinical manifestations in PD patients. We consecutively recruited 61 idiopathic PD patients and 47 healthy controls (HC) who were evaluated by clinical scales, genotyped for variant Ser9Gly in DRD3, and underwent resting-state functional magnetic resonance imaging. Based on DRD3 Ser9Gly polymorphism, PD patients and HCs were divided into four subgroups. Then, two-way analysis of covariance (ANCOVA) was applied to investigate main effects and interactions of PD and DRD3 Ser9Gly polymorphism on the brain function via amplitude of low-frequency fluctuations (ALFF) approach. The association between DRD3 Ser9Gly-modulated significantly different brain regions, and clinical manifestations were detected by Spearman's correlations. PD patients exhibited decreased ALFF values in the right inferior occipital gyrus, lingual gyrus, and fusiform gyrus. A significant difference in the interaction of “groups × genotypes” was observed in the right medial frontal gyrus. The ALFF value of the cluster showing significant interactions was positively correlated with HAMD-17 scores (*r*=0.489, *p*=0.011) and anhedonia scores (*r*=0.512, *p*=0.008) in PD patients with the Ser/Gly or Gly/Gly genotypes. Therefore, D3 gene Ser9Gly polymorphism might be associated with the severity of depression characterized by anhedonia in PD patients.

## 1. Introduction

The characteristic of PD neuropathology is a selective loss of dopaminergic neurons in the substantia nigra pars compacta [1], and orally administered levodopa is one of the standard treatments of PD. As dopamine receptors provide vital determinants of dopamine function [2], there is no doubt that polymorphisms in dopamine receptor genes have an effect on PD. There are five dopamine receptors (DRs) subtypes, of which DR1 and DR5 are composed of D1-like receptors, while DR2, DR3, and DR4 consist of D2-like receptors [3]. The most frequently studied variant point of the DRD3 is DRD3 Ser9Gly polymorphism [4]. Although the emerging fact demonstrates that DRD3 Ser9Gly polymorphism is closely associated with the clinical manifestations [4–8] and the treatment of PD [9, 10], the results were inconsistent. So intensive studies are needed to explore the role of DRD3 Ser9Gly polymorphism in PD.

Imaging genetics is an integrated research method which uses neuroimaging and genetics to evaluate the impact of genetic variation on brain function and structure [11], which has been widely used to investigate the effect of gene in several diseases such as PD [12], amnestic mild cognitive impairment (aMCI) [13], and major depressive disorder (MDD) [14]. Moreover, resting-state functional magnetic resonance imaging (rs-fMRI) allows studying spontaneous brain activity in absence of task, which records changes of Blood Oxygenation Level-Dependent (BOLD) signal [15]. Therefore, rs-fMRI could be used as a platform to evaluate the gene function in the brain. Amplitude of low-frequency fluctuation (ALFF) which detects the spontaneous amplitude of low-frequency (0.01–0.08 Hz) BOLD signal is one of the most reliable and reproducible rs-fMRI parameters and effectively reflects the level of regional functional neural activity [16].

In this study, we were the first to use ALFF to investigate the potential effect of DRD3 Ser9Gly polymorphism on modulating resting-state brain function and associative clinical manifestations in PD patients.

## 2. Materials and Methods

### 2.1. Study Participants

According to the UK Parkinson's Disease Society Brain Bank Research criteria [17], 61 well-characterized idiopathic PD patients were consecutively recruited from the First Affiliated Hospital of Nanjing Medical University between June 2017 and February 2018. PD patients who were diagnosed of neurological diseases other than PD, other forms of parkinsonism such as atypical parkinsonism and secondary parkinsonism or combined with severe cognitive impairment (Mini-Mental State Exam (MMSE) score < 24) [18], were ruled out in this study. The diagnosis was confirmed by two experienced neurologists, Kezhong Zhang and Yongsheng Yuan, to ensure the reliability of the study. Moreover, we consecutively recruited 47 age- and gender-matched healthy controls without neurological disorders (including movement disorders, parkinsonism, neurodegenerative diseases such as dementias), psychological disorders, a family history of PD, cognitive impairment, or imaging abnormalities from local individuals who volunteered to participate in scientific studies by advertising. Participants with sever acute or chronic diseases, contraindications for MRI scans, or a recent history of using antidepressant, anxiolytic, or antipsychotic drugs were also excluded. All participants were East Asian individuals and lived in Jiangsu Province, China. To minimize conceivable pharmacological impacts on neural activity, MRI scans and clinical examinations were performed during off-state (at least 12-hour withdrawal of pharmacologic treatment for PD) in PD patients. This study was approved by the Ethics Committee of the First Affiliated Hospital of Nanjing Medical University, and all participants provided us with written informed consent before participating in the experiment.

### 2.2. Clinical Assessment

The third part of the Unified Parkinson's Disease Rating Scale (UPDRS-III) [19] and the Hoehn and Yahr (H&Y) staging were applied to evaluate the motor severity in the PD group. Moreover, we used Tinetti Mobility Test [20] to assess the balance and gait of PD patients. Furthermore, we detected the severity of fatigue, depression, anxiety, and apathy in PD patients using the Fatigue Severity Scale (FSS) [21], the Hamilton Depression Scale (HAMD) [22], the Hamilton Anxiety Scale (HAMA) [23], and the Modified Apathy Evaluation Scale (MAES) [24], respectively. Cognitive functioning and executive functioning of PD patients were evaluated by the Mini-Mental State Examination (MMSE) and the Frontal Assessment Battery (FAB) [25]. Epworth Sleepiness Scale (ESS) [26] was used to assess the sleepiness of PD patients. As previous studies reported that DRD3 Ser9Gly polymorphism was associated with anhedonia in patients with major depressive disorder, we decided to measure anhedonia in PD patients to further explore the role of DRD3 Ser9Gly polymorphism in PD [27]. Anhedonia score was measured by a single anhedonia item in the HAMD scale. This item measured a dimensional construct including desire, effort, and consummatory pleasure [22]. Levodopa-equivalent daily dose (LEDD) was calculated according to the widely accepted method [28].

### 2.3. DRD3 Genotyping

The analysis was performed in a blinded manner by experts who had no knowledge of all participants. Ten milliliters of peripheral blood from the antecubital vein was collected to extract DNA. Then, DNA was genotyped for DRD3 rs6280 (Ser9Gly). MassARRAY TYPER 4.0 software (Agena, Inc) was used to process and analyze the data. *χ*^2^ test was used to check Hardy–Weinberg equilibrium. Then, the subjects were divided into different subgroups (Ser/Ser carriers and Gly carriers).

### 2.4. Image Acquisition

All participants were scanned using a 3.0 Tesla Siemens MAGNETOM Verio whole-body MRI system (Siemens Medical Solutions, Germany) which was equipped with eight-channel and phase-array head coils by experienced doctors from the radiology department. Tight foam padding was used to minimize head motion. As the scanner made big noise, earplugs were used. Participants were instructed to keep awake, eyes closed and motion less. They also tried their best not to think about anything. Three-dimensional T1-weighted anatomical images were obtained using a volumetric 3D magnetization-prepared rapid gradient-echo (MP-RAGE) sequence (repetition time (TR) = 1900 ms, echo time (TE) = 2.95 ms, flip angle (FA) = 9°, slice thickness = 1 mm, slices = 160, field of view (FOV) = 230 × 230 mm^2^, matrix size = 256 × 256, and voxel size = 1 × 1 × 1 mm^3^). Resting-state functional images were obtained using an echo-planar imaging (EPI) sequence (TR = 2000 ms, TE = 21 ms, FA = 9°, FOV = 256 × 256 mm^2^, in-plane matrix = 64 × 64, slices = 35, slice thickness = 3 mm, no slice gap, voxel size = 3 × 3 × 3 mm^3^, and total volumes = 240).

### 2.5. MRI Data Preprocessing

Statistical Parametric Mapping (SPM8, http://www.fil.ion.ucl.ac.uk), the data processing assistant for resting-state fMRI (DPARSF, http://www.restfmri.net), and MATLAB were used to perform MRI data preprocessing. The preprocessing of MRI data included exclusion of the first ten volumes of each functional time course, slice timing correction, head motion correction, regressing out of nuisance variables, spatial normalization, resampling of images into a spatial resolution of 3 × 3 × 3 mm^3^, and spatial smoothening with a Gaussian kernel (full width at half-maximum = 4 × 4 × 4 mm^3^). Nuisance variable included the white matter, cerebrospinal fluid, and Friston 24-parameter head motion. Participants with head motion of more than 2.0 mm of translation or 2.0° of rotation during the course of the scan were ruled out. In order to minimize temporal drifts and white noise, the resulting data were temporally bandpass filtered (0.01–0.08 Hz).

### 2.6. ALFF Analysis

ALFF values were calculated in the frequency range of 0.01–0.08 Hz using DPARSF. Briefly, the calculation steps included converting all voxels from the time domain to the frequency domain and averaging the square root of the power spectrum between 0.01 Hz and 0.08 Hz.

### 2.7. Statistical Analysis

The analysis of demographic and neuropsychological data was performed using the SPSS 20.0 statistical analysis software (SPSS Inc. Chicago, IL, USA). Continuous variables and categorical variables were shown as median (range) and percentage, respectively. Comparisons of demographic data (gender, age, and education) among groups were performed using chi-square tests and two-way analysis of variance (ANOVA). As many neuropsychological data were not normally distributed, Mann–Whitney *U* tests were used to compare clinical data across genotypes within the PD group. Chi-square test was used to test the Hardy–Weinberg Equilibrium (HWE) of the genotype frequencies. A significant threshold was set at *p* < 0.05.

A two-way analysis of variance (ANOVA: groups × genotypes; groups: PD and healthy controls; genotypes: Ser/Ser carriers and the Gly carriers) with gender, age, and education as nuisance variables was performed to determine the effects of group and genotype on ALFF (voxel-level *p* < 0.001, cluster size > 10 voxels, corresponding to a corrected *p* < 0.001 as based on Monte Carlo simulations) (http://afni.nimh.nih.gov/pub/dist/doc/manual/AlphaSim.pdf). Post hoc tests were performed to further explore some statistical differences. Finally, we explored the relationship between the neuropsychological test scores and the ALFF values of the clusters which showed significant effect of groups or the interaction between groups and genotypes using Spearman's Rho to detect the correlation significance.

## 3. Results

### 3.1. Demographic and Neuropsychological Characteristics

The genotype frequencies for DRD3 Ser9Gly in the PD group and the healthy control group are displayed in Table 1, which did not deviate from Hardy–Weinberg equilibrium (PD group: *χ*^2^ = 2.181, *P*=0.140; control group: *χ*^2^ = 0.084, *P*=0.772). Other demographic and neuropsychological characteristics of participants are displayed in Table 2. There was no significant effect of diagnosis, genotype, or interaction between diagnosis and genotype for gender, age, or education. Moreover, there was no significant difference in clinical data including disease duration, H&Y stage, LEDD, UPDRS-III, MMSE, FAB, Tinetti Balance, Tinetti Gait, FSS, HAMA, Apathy, ESS between Ser/Ser carriers, and Gly carriers in the PD group. Nevertheless, the HAMD scores in PD with the Ser/Gly or Gly/Gly genotypes were significantly higher than PD with the Ser/Ser genotypes (*P*=0.012). Furthermore, the anhedonia scores in PD with the Ser/Gly or Gly/Gly genotypes were significantly higher than PD with the Ser/Ser genotypes (*P*=0.004).

### 3.2. ALFF Data

**Figure 1:**
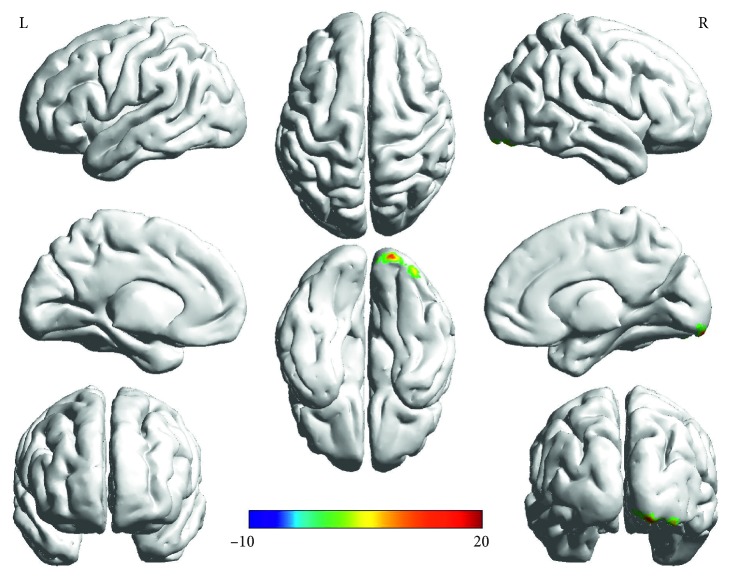
Main effect of groups on ALFF in PD and HC found in the right occipital lobe (right inferior occipital gyrus/lingual gyrus/fusiform gyrus). The finding was obtained via two-way factorial analysis of covariance (ANCOVA: groups × genotypes; groups: PD and HC, genotypes: Ser/Ser carriers and the Gly carriers) adjusting for age, gender, and education. A corrected threshold by Monte Carlo simulation was set at *P* < 0.001. The color bar indicates the *F* values from ANCOVA. Abbreviations: PD, Parkinson's disease; HC, healthy control; ALFF, amplitude of low-frequency fluctuations; R, right; L, left.

**Figure 2:**
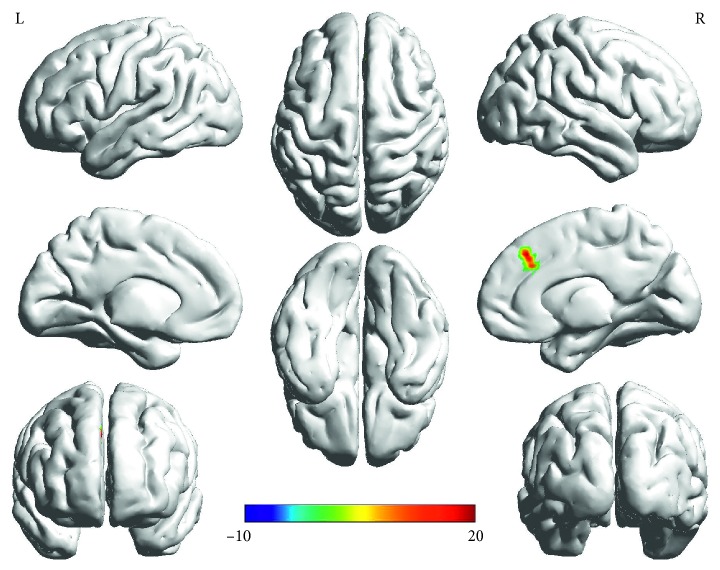
The interaction between groups and genotypes found in the right medial frontal gyrus. The finding was obtained via two-way factorial analysis of covariance (ANCOVA: groups × genotypes; groups: PD and HC, genotypes: Ser/Ser carriers and the Gly carriers) adjusting for age, gender, and education. A corrected threshold by Monte Carlo simulation was set at *P* < 0.001. The color bar indicates the *F* values from ANCOVA. Abbreviations: PD, Parkinson's disease; HC, healthy control; ALFF, amplitude of low-frequency fluctuations; DRD3, dopamine receptor D3 gene; R, right; L, left.

Two-way ANOVA (Table 3): Main effect of group (PD, HC) was found in right occipital lobe (right inferior occipital gyrus/lingual gyrus/fusiform gyrus) (*F* = 23.44, *p* < 0.001, corrected) (Figure 1). The “groups × genotypes” interaction was observed in the right frontal lobe (right medial frontal gyrus) (*F* = 23.02, *p* < 0.001, corrected) (Figure 2). However, the main effect of gene (the Ser/Gly or Gly/Gly genotypes, the Ser/Ser genotypes) was not observed.

**Figure 3:**
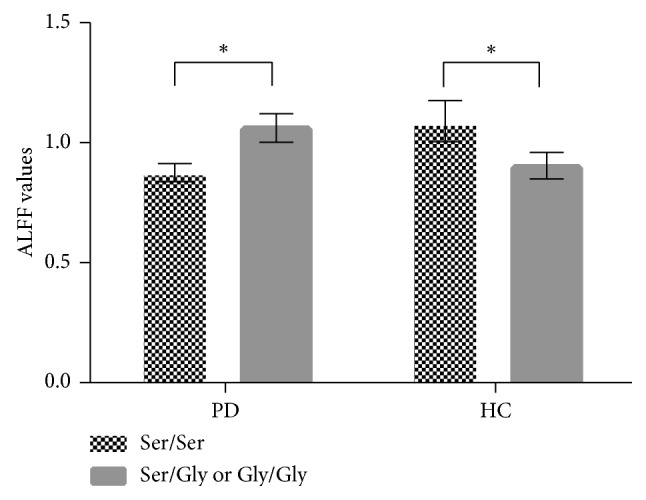
Post hoc tests of the interaction between groups and genotypes. Post hoc tests were corrected by Bonferroni correction with a significant different *p* < 0.0083 (0.05/6 (number of pair-comparisons)). ALFF values in the right medial frontal gyrus were increased in the PD group with the Ser/Gly or Gly/Gly genotypes when compared to the Ser/Ser genotype, while it was decreased in the control group with the Ser/Gly or Gly/Gly genotypes when compared to the Ser/Ser genotype. Abbreviations: PD, Parkinson's disease; HC, healthy control; ALFF, amplitude of low-frequency fluctuations; DRD3, dopamine receptor D3 gene. ^*∗*^*p* < 0.01.

Post hoc tests: Post hoc tests were corrected by Bonferroni correction with a significant different *p* < 0.0083 (0.05/6 (number of pair-comparisons)). ALFF values in the brain regions of the right medial frontal gyrus were increased in the PD group with the Ser/Gly or Gly/Gly genotypes when compared to the Ser/Ser genotype (*p* ≤ 0.001) while decreased in the control group with the Ser/Gly or Gly/Gly genotypes when compared to the Ser/Ser genotype (*p*=0.001). ALFF values in the right medial frontal gyrus were lowest in PD patients with the Ser/Ser genotype among four subgroups (Figure 3).

### 3.3. Correlation Analysis

**Figure 4:**
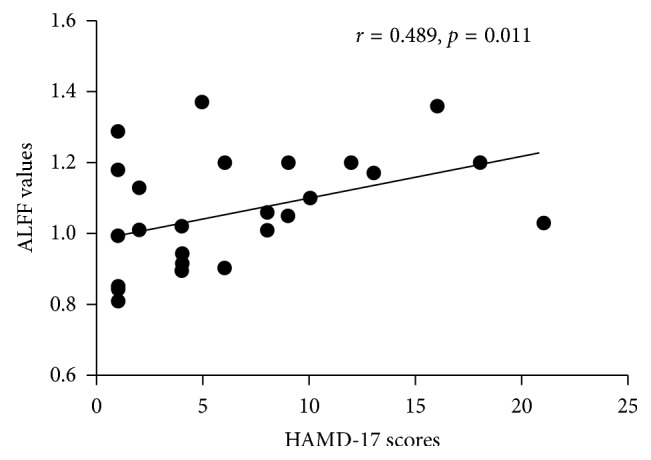
Correlation analysis between ALFF values in the right medial frontal gyrus and HAMD-17 scores. ALFF values in the right medial frontal gyrus affected by the interaction between groups and genotypes were positively correlated with HAMD-17 scores (*r* = 0.489, *p*=0.011) in the Gly carriers in the PD group. Abbreviations: PD, Parkinson's disease; ALFF, amplitude of low-frequency fluctuations; HAMD, Hamilton Depression Scale.

**Figure 5:**
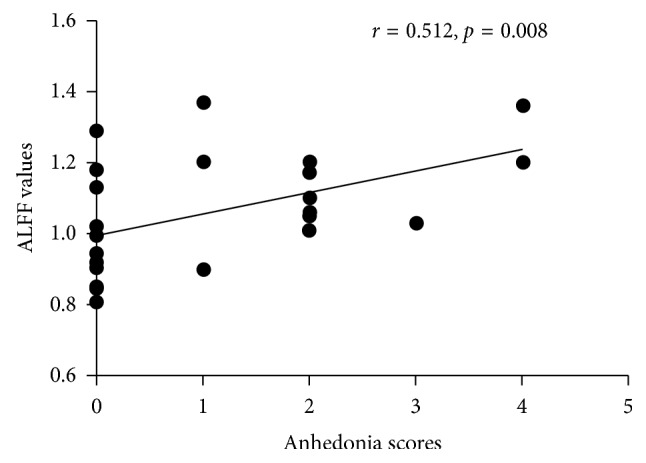
Correlation analysis between ALFF values in the right medial frontal gyrus and anhedonia scores. ALFF values in the right medial frontal gyrus affected by the interaction between groups and genotypes were negatively correlated with anhedonia scores (*r* = 0.512, *p*=0.008) in the Gly carriers in the PD group. Abbreviations: PD, Parkinson's disease; ALFF, amplitude of low-frequency fluctuations.

ALFF values in the right medial frontal gyrus affected by interactions between groups and genotypes were positively correlated with HAMD-17 scores (*r* = 0.489, *p*=0.011) (Figure 4) and anhedonia scores (*r* = 0.512, *p*=0.008) (Figure 5) in the Gly carriers in the PD group, while it had no association with HAMD-17 scores or anhedonia scores in the other group. There was no significant correlation between ALFF values in the right medial frontal gyrus and UPDRS-III, MMSE, FAB, Tinetti Balance, Tinetti Gait, FSS, HAMA, Apathy, and ESS scores (*p* > 0.05) within each group.

## 4. Discussion

This study was the first to investigate the potential effect of DRD3 Ser9Gly polymorphism on modulating resting-state brain function in idiopathic PD patients and healthy controls. Moreover, we used sensitive clinical scales to carry out detailed clinical assessments, so we could further explore the relationship between DRD3 Ser9Gly polymorphism and associative clinical manifestations in PD patients.

DRD3 is located in 3q13.31 and mainly expresses in the ventral striatum and the globus pallidus where it regulates both dopamine release and clearance from extracellular space by the dopamine transporter (DAT) [29]. The DR3 is more selectively associated with the limbic areas of the brain which receives its dopamine input from the ventral tegmental area and plays an important role in cognitive, emotional, and endocrine functions [9]. Recently, some studies reported that DRD3 Ser9Gly polymorphism was associated with impulse-control disorders in PD patients, but their results were inconsistent [4–6]. Rajan et al. found that DRD3 Ser9Gly polymorphism is associated with aberrant decision-making under uncertainty in PD patients without active impulse-control disorders [7]. Goetz et al. observed that there was a higher frequency of the Gly allele in the hallucinators [8], while Wang et al. reported that no significant difference was found between hallucinators and nonhallucinators in the DRD3 Ser9Gly genotypic or allelic distributions [2]. Differences in the methods used for the assessment, the size of the samples studied, and the severity of the disease may lead to the discrepancy in the results. Moreover, the Gly/Gly group and the Gly/Ser group respond worse to pramipexole than the Ser/Ser group [9, 10]. Recent large meta-analyses suggest that DRD3 Ser9Gly polymorphism had no relationship to increased susceptibility to PD [30], indicating that DRD3 Ser9Gly might be a minor gene locus in the occurrence of sporadic PD. Hence, the main effect of DRD3 Ser9Gly in PD is alterations in some brain regions function instead of the susceptibility to disease. That is to say, exploring the association between genetic variants and clinical manifestations is more meaningful than exploring the association between genetic variants and the susceptibility to disease. Imaging genetics is a new method to study association between genetic variants and imaging phenotypes and further explore the association between genetic variants and clinical manifestations.

Our analysis in main effect of genotypes showed that there was no difference between Ser/Ser carriers and the Gly carriers in resting-state brain function, regardless of the disease status. Interestingly, a significant difference in the “groups × genotypes” interaction was observed in the right medial frontal gyrus, which demonstrated that there were differential effects of DRD3 Ser9Gly polymorphism in PD patients and healthy controls. Hence, both genetic factors and physical conditions should be taken into considerations to clarify a pathogenic role of DRD3 Ser9Gly polymorphism in PD. What is more, ALFF values in the right medial frontal gyrus were lower in Ser/Ser carriers than the Gly carriers in the PD group, while ALFF values in the right medial frontal gyrus were lower in the Gly carriers than Ser/Ser carriers in the HC group. This phenomenon indicated that PD patients with the Ser/Gly or Gly/Gly genotypes might potentially show evidence of functional brain abnormalities. Moreover, the ALFF value of the cluster showing significant interactions had relationships to HAMD-17 scores and anhedonia scores in PD patients with the Ser/Gly or Gly/Gly genotypes. HAMD-17 is a 17-item clinician-rated instrument developed to quantify the severity of depression, which has become one of the most widely used outcome measures in depression [31]. Therefore, DRD3 Ser9Gly polymorphism might be associated to the severity of depression characterized by anhedonia in PD patients with the Ser/Gly or Gly/Gly genotypes.

It has been widely reported that the Gly carriers are more common and anhedonic than Ser/Ser carriers among patients with major depressive disorder [27, 32, 33], indicating that DRD3 Ser9Gly polymorphism is implicated in the pathogenesis of depression. Compared with the Ser-9 variant, the Gly-9 variant has a significantly higher binding affinity for dopamine and exhibits higher cAMP inhibition and MAPK signal duration than Ser-9 variant [34], which attenuates the function of the D3 receptor [9]. The higher affinity of the glycine autoreceptor is linked to the decrease of the extrasynaptic dopamine concentration under conditions of tonic dopamine release [33], which leads to the change of the neural circuit underlying reward-related mechanisms (the cortico-limbic-striatal circuits) [35]. As shown in Figure 2, the activated brain region of the “groups × genotypes” interaction in the right medial frontal gyrus is located in the right medial prefrontal cortex (mPFC). In both humans and animals, mPFC is a critical node in a distributed neural network which regulates many cognitive and limbic functions [36]. Previous studies suggest that the chronic mPFC overactivity stably suppressed natural reward-motivated behaviors and induced specific new brainwide functional interactions, which predicted the degree of anhedonia in individuals [37]. Moreover, mPFC is an important part of the reward-related neural circuit [38]. Therefore, we speculated that PD patients with the Ser/Gly or Gly/Gly genotypes had a deficiency in the reward-related neural circuit which led to anhedonia.

It must be acknowledged that our study had some limitations. Firstly, we integrated individuals with the Ser/Gly or Gly/Gly genotypes into a single group because of small sample size, which might have an adverse effect on the observation of “gene effect”. Secondly, our study was a cross-sectional study and longitudinal studies are needed to further assess the progress of disease in the same participants. Thirdly, we ignored dopamine receptor genes other than DRD3 and did not study the gene-gene interaction. Fourthly, our study did not include some essential clinical symptoms such as impulse-control disorders, aberrant decision-making, hallucinations, and behavioral addictions which might be associated with DRD3 Ser9Gly polymorphism in PD patients [4–8]. Fifthly, the right medial frontal gyrus is engaged in many other cognitive functions [36, 39]. We excluded patients with cognitive impairment due to a consideration that their self-reporting on the questionnaire was likely unreliable. As a result, we could not carry on cognitive-related tests to further judge whether ALFF values in the right medial frontal gyrus affected has relationship to cognitive functions or not. Last but not least, we chose to evaluate anhedonia by one item of the HAMD rather than a specific questionnaire validated in PD patients, which was inaccurate. Therefore, a longitudinal research which has a larger sample size studies more clinical manifestations, includes patients with cognitive impairment, evaluates anhedonia by a specific questionnaire, and uses multimodal techniques is needed in the future.

## 5. Conclusions

The present study was the first time to demonstrate that the right medial frontal gyrus activation related to DRD3 Ser9Gly polymorphism is associated with the occurrence and the severity of depression in PD, in particular in a type of depression characterized by anhedonia, which might play a key role in the pathogenesis of depression in PD. Therefore, the right medial frontal gyrus activation related to DRD3 Ser9Gly polymorphism could be a biomarker for the occurrence and the severity of depression in PD. However, whether new biological measure could be invented to treat depression in PD patients with the Ser/Gly or Gly/Gly genotypes according to our findings or not needs further studies to judge. Furthermore, imaging genetics as a new promising approach was available to study the relationship between gene polymorphisms and the complicated clinical manifestations in PD.

## Figures and Tables

**Table 1:** Genotype frequencies for DRD3 gene Ser9Gly in PD group and HC group.

Genotype	PD (frequency)	HC (frequency)	Total (frequency)
Ser/Ser	35 (57.4%)	20 (42.6%)	55 (50.9%)
Ser/Gly	25 (41.0%)	22 (46.8%)	47 (43.5%)
Gly/Gly	1 (1.6%)	5 (10.6%)	6 (5.6%)

Genotype frequencies for DRD3 Ser9Gly in the PD group (*χ*^2^ = 2.181, *P*=0.140) and the HC group (*χ*^2^ = 0.084, *P*=0.772) did not deviate from Hardy–Weinberg equilibrium. Abbreviations: DRD3, dopamine receptor D3 gene; PD, Parkinson's disease; HC, healthy control.

**Table 2:** Demographic data of participants.

Items	PD		HC		*p*
	Ser/Ser (*n*=35)	Ser/Gly or Gly/Gly (*n*=26)	Ser/Ser (*n*=20)	Ser/Gly or Gly/Gly (*n*=27)	
Gender (male/female)	22/13	19/7	10/10	19/8	0.372^a^
Age (years)	68 (44–81)	65.5 (49–88)	64.5 (52–72)	63 (55–72)	0.119^b^
Education (years)	11 (6–20)	12 (5–17)	12 (5–22)	12 (5–16)	0.845^b^
Disease duration (years)	4 (0.25–13)	3.125 (0.5–20)	NA	NA	0.930^c^
H&Y stage	2 (1–3)	2 (1–3)	NA	NA	0.424^c^
LEDD (mg/day)	375 (0–1025)	400 (0–837.5)	NA	NA	0.965^c^
UPDRS-III	22 (8–48)	22 (5–40)	NA	NA	0.815^c^
MMSE	29 (26–30)	29 (24–30)	NA	NA	0.477^c^
FAB	16 (10–18)	16 (10–18)	NA	NA	0.489^c^
Tinetti Balance	14 (4–16)	14 (3–16)	NA	NA	0.555^c^
Tinetti Gait	9 (2–12)	8.5 (4–12)	NA	NA	0.959^c^
FSS	28 (9–55)	26 (8–57)	NA	NA	0.569^c^
HAMD-17	2 (0–16)	4.5 (1–21)	NA	NA	0.012^*c,∗*^
HAMA	7 (1–18)	9 (2–24)	NA	NA	0.270^c^
Apathy	14 (2–36)	17.5 (1–32)	NA	NA	0.310^c^
ESS	5 (0–14)	4.5 (0–11)	NA	NA	0.282^c^
Anhedonia score^&^	0 (0–2)	1 (0–4)	NA	NA	0.004^*c,∗*^

Abbreviations: PD, Parkinson's disease; HC, healthy control; H&Y stage, Hoehn and Yahr stage; LEDD, levodopa-equivalent daily dose; UPDRS, Unified Parkinson's Disease Rating Scale; MMSE, Mini-Mental State Examination; FAB, Frontal Assessment Battery; FSS, Fatigue Severity Scale; HAMD, Hamilton Depression Scale; HAMA, Hamilton Anxiety Scale; ESS, Epworth Sleepiness Scale; NA, not applicable. Values except gender were expressed as median (range). ^a^Chi-square tests. ^b^Two-way analysis of variance (ANOVA). ^c^Mann–Whitney *U* tests. ^&^Anhedonia score was measured by a single anhedonia item in the HAMD scale. ^*∗*^*P* < 0.05 was considered significant.

**Table 3:** Groups × genes ANOVA of ALFF.

Brain region	Peak MNI coordinates (mm)			Peak *F* value	Clusters size (mm^3^)
*Main effect of groups*					
Right inferior occipital gyrus/lingual gyrus/fusiform gyrus	36	−90	−21	23.44	1377

*Main effect of genotypes*					
None					
Groups × genotypes interaction					
Right medial frontal gyrus	6	33	42	23.02	486

Two-way factorial analysis of covariance (ANCOVA: groups × genotypes; groups: PD and HC, genotypes: Ser/Ser carriers and the Gly carriers) was performed, adjusting for age, gender, and education. A corrected threshold by Monte Carlo simulation was set at *P* < 0.001; Abbreviations: PD, Parkinson's disease; HC, healthy control; ALFF, amplitude of low-frequency fluctuations; MNI, Montreal Neurological Institute.

## Data Availability

The data are available to qualified investigators on request to the corresponding and senior authors.
